# Psychometric properties of the parent-report version of the Strengths and Difficulties Questionnaire (SDQ) in a clinical population of Latvian children and adolescents

**DOI:** 10.1192/j.eurpsy.2023.850

**Published:** 2023-07-19

**Authors:** N. Bezborodovs, A. Kocane, E. Rancans, A. Villerusa

**Affiliations:** 1 Department of Psychiatry and Narcology, Riga Stradins University; 2 Child psychiatry clinic, Children’s clinical university hospital; 3 Department of Public Health and Epidemiology, Riga Stradins University, Riga, Latvia

## Abstract

**Introduction:**

Screening instruments can be crucial in child and adolescent mental healthcare practice by allowing to triage the patient flow in a limited resource setting and help in clinical decision making. However, for a screening procedure to work, we must be sure that the screening tools used have reasonable validity and clinical utility in the population they are used in.

**Objectives:**

Our study aimed to examine the psychometric and predictive properties of the parent-report version of the Strengths and Difficulties Questionnaire (SDQ), with the application of the original UK-based scoring algorithm, in a clinical psychiatric population sample of Latvian children and adolescents.

**Methods:**

363 outpatients aged 2 to 17 years from two outpatient child psychiatry centres in Latvia were screened with the parent-report version of the SDQ and assigned clinical psychiatric diagnoses. The basic psychometric properties, and ability of the SDQ to predict the clinical diagnosis in major diagnostic groups (emotional, conduct, hyperactivity, and developmental disorders) was assessed.

**Results:**

Most of the study participants were male (n=230, 63%). The mean age was 9,28 (SD=3,82) years for males and 10,93 (SD=4,11) years for females.

Emotional problems, hyperactivity, and prosocial subscales of the SDQ, as well as the externalising and total difficulties scales, demonstrated acceptable internal consistency (Cronbach’s alfa > 0,7). The results for the conduct problems and internalising difficulties scales were also close to being on the acceptable level (0,68 and 0,69 respectively). The peer problems subscale was the only SDQ scale with poor internal consistency (0,57).

The subscales of the parent-report SDQ showed significant correlation with the corresponding clinical diagnoses. The sensitivity and specificity of appropriate subscales of the parent-report SDQ were 67% CI [0,57,0,77] and 57% CI [0,50, 0,64] for any emotional disorder, 78% CI [0,67, 0,89] and 57% CI [0,50, 0,64] for any conduct disorder, 65% CI [0,55, 0,75] and 78% CI [0,73, 0,83] for the hyperkinetic disorder, 72% CI [0,63, 0,81] and 44% CI [0,36, 0,52] for developmental disability.

Overall, none of the subscales of the SDQ has reached the interval of potential usefulness for clinical decision-making in specialized psychiatric settings, based on the positive likelihood ratio, negative likelihood ratio and diagnostic odds ratio estimates.

**Conclusions:**

We suggest the SDQ rather be used in primary healthcare settings, where it can be an essential tool to help family physicians recognise children needing further specialised psychiatric evaluation. There is a need to assess the psychometric properties and validate the SDQ in a larger populational sample in Latvia, determine the population-specific cut-off scores, and reassess the performance of the scale in primary healthcare practice.

**Disclosure of Interest:**

None Declared

